# An improved multivariate model that distinguishes COVID-19 from seasonal flu and other respiratory diseases

**DOI:** 10.18632/aging.104132

**Published:** 2020-10-21

**Authors:** Xing Guo, Yanrong Li, Hua Li, Xueqin Li, Xu Chang, Xuemei Bai, Zhanghong Song, Junfeng Li, Kefeng Li

**Affiliations:** 1 Department of Radiology, Heping Hospital Affiliated to Changzhi Medical College, Shanxi 046000, China; 2 Department of Pharmacy, Changzhi Medical College, Shanxi 046000, China; 3 Department of Respiratory Medicine, Third Hospital of Linfen, Shanxi 041000, China; 4 Department of Respiratory Medicine, Jincheng General Hospital, Shanxi 048006, China; 5 Graduate School of Changzhi Medical College, Shanxi 046000, China; 6 Department of Nephrology, Jiexiu People’s Hospital, Shanxi 032000, China; 7 Department of Nephrology, Fenyang Hospital, Shanxi 032200, China; 8 School of Medicine, University of California, San Diego, CA 92093, USA

**Keywords:** COVID-19, multi-feature, influenza, random forest, diagnostic model

## Abstract

COVID-19 shared many symptoms with seasonal flu, and community-acquired pneumonia (CAP) Since the responses to COVID-19 are dramatically different, this multicenter study aimed to develop and validate a multivariate model to accurately discriminate COVID-19 from influenza and CAP. Three independent cohorts from two hospitals (50 in discovery and internal validation sets, and 55 in the external validation cohorts) were included, and 12 variables such as symptoms, blood tests, first reverse transcription-polymerase chain reaction (RT-PCR) results, and chest CT images were collected. An integrated multi-feature model (RT-PCR, CT features, and blood lymphocyte percentage) established with random forest algorism showed the diagnostic accuracy of 92.0% (95% CI: 73.9 - 99.1) in the training set, and 96. 6% (95% CI: 79.6 - 99.9) in the internal validation cohort. The model also performed well in the external validation cohort with an area under the receiver operating characteristic curve of 0.93 (95% CI: 0.79 - 1.00), an F1 score of 0.80, and a Matthews correlation coefficient (MCC) of 0.76. In conclusion, the developed multivariate model based on machine learning techniques could be an efficient tool for COVID-19 screening in nonendemic regions with a high rate of influenza and CAP in the post-COVID-19 era.

## INTRODUCTION

Coronavirus disease 2019 (COVID-19) is caused by severe acute respiratory syndrome coronavirus 2 (SARS-CoV-2), which is a beta coronavirus. COVID-19 has spread globally since the first case reported in Wuhan, China, at the end of December 2019 [1]. As of April 16, 2020, COVID-19 has affected 185 countries and territories around the world, with 2,224,426 confirmed cases [2]. In addition to the ongoing COVID-19 pandemic, many countries are in the flu season. Patients with COVID-19 share many similarities in clinical symptoms and imaging characteristics with those with seasonal flu and pneumonia caused by other common respiratory pathogens [3]. Therefore, differentiating patients with COVID-19 from those with other seasonal respiratory diseases in a hospital setting is critical.

The accurate diagnosis of COVID-19 is challenging. Currently, reverse transcription-polymerase chain reaction (RT-PCR)-based analysis of nasopharyngeal swabs is the reference standard. However, the diagnostic sensitivity of RT-PCR testing is less optimal, and the magnitude of risk from false-negative test results is substantial [4]. Even though the utility of chest computed tomography (CT) for the detection of COVID-19 has been demonstrated in a few recent publications, many methodological flaws were present in these studies [5]. Additionally, several parameters in routine blood tests might be useful in predicting the severity of COVID-19 [6]. A new classification model that combines the advantages of various techniques is urgently needed.

Random forest (RF) has important advantages over other machine learning algorithms in terms of handling multidimensional and nonlinear biological data, the opportunity for efficient parallel processing, and its robustness to noise [7].

In this study, we developed an integrated multi-feature model based on RF to differentiate COVID-19 from seasonal flu and pneumonia caused by other common respiratory viruses. The performance of the model was validated using both an internal validation set and an external validation cohort from another hospital.

## RESULTS

### Patient characteristics

**Figure 1:**
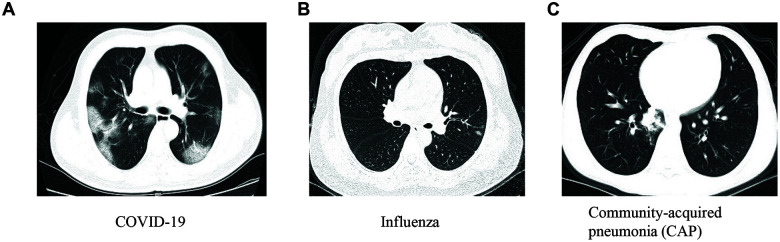
****The representative chest images for patients with COVID-19 (**A**), influenza (**B**) and community-acquired pneumonia (CAP) (**C**).

This study was conducted in a region outside of Wuhan, the epicenter of the COVID-19 outbreak. Out of 50 suspected patients enrolled in the discovery and internal validation cohorts, 8 had COVID-19, 8 had seasonal flu (influenza), and 34 had community-acquired pneumonia (CAP) (Table 1). There were no significant differences in characteristics among the three groups, including age, sex, clinical symptoms, WBC, and LYC. Compared to those with influenza and CAP, patients with COVID-19 had significantly lower LYP values (*P* < 0.001). Representative chest CT images for COVID-19, influenza, and CAP are shown in Figure 1. The external validation cohort from another hospital included 11 COVID-19 patients, 20 with influenza, and 24 with CAP (Table 2).

**Table 1:** Patient characteristics in the discovery and internal validation cohorts.

**Characteristics**	**COVID-19 (n = 8)**	**Influenza (n = 8)**	**CAP (n = 34)**	***P* value**
Age (years)	25.1 (24.2 - 62.5)	29.5 (25.6 - 54.3)	31 (23.1 - 56.4)	0.93
Male, n(%)	4 (50%)	4 (50%)	22 (64.5%)	0.36
Fever, n (%)	2 (25%)	4 (50%)	15 (44.1%)	0.43
Cough, n (%)	2 (25%)	1 (12.5%)	12 (35.3%)	0.37
Sore throat, n (%)	2 (25%)	1 (12.5%)	3 (8.8%)	0.22
Fatigue, n (%)	2 (25%)	2 (25%)	4 (11.7%)	0.27
WBC (10^9^/L)	5.3 (3.6 - 6)	4.9 (3.2 - 6.2)	5.5 (4.1 - 6.5)	0.65
Lymphocyte count (10^9^/L)	0.93 (0.76 - 1.35)	1.4 (0.9 - 1.9)	1.5 (1.3 - 2.1)	0.08
Lymphocyte percentage (%)	14.2 ± 6.3	36.4 ± 7.1	33.9 ± 10.1	<0.001

The data are presented as the median and interquartile range (IQR), n (%) or mean and standard deviation (SD) For categorical data, chi-square test was used, and for continuous variables, Kruskal-Wallis was applied. WBC: white blood cell count.

**Table 2:** Patient characteristics in the external validation cohort enrolled from another hospital.

**Characteristics**	**COVID-19 (n = 11)**	**Influenza (n = 20)**	**CAP (n = 24)**	***P* value**
Age (years)	31.0 (25.0 - 50.0)	32.0 (26.5 - 52.3)	39.0 (27 - 48)	0.95
Male, n (%)	6 (54.5%)	12 (60.0%)	15 (62.5%)	0.66
Fever, n (%)	9 (81.8%)	13 (65.0%)	20 (83.3%)	0.65
Cough, n (%)	3 (27.3%)	9 (45.0%)	11 (45.8%)	0.36
Sore throat, n (%)	1 (9.1%)	3 (15.0%)	2 (8.3%)	0.81
Fatigue, n (%)	1 (9.1%)	6 (30.0%)	4 (16.7%)	0.86
WBC (10^9^/L)	6.5 (5.1 – 8.8)	7.7 (5.5 - 9.8)	6.8 (5.3 - 8.2)	0.32
Lymphocyte count (10^9^/L)	1.2 (1.0 - 1.9)	1.3 (0.9 - 2.1)	1.7 (1.3 - 2.3)	0.03
Lymphocyte percentage (%)	13.1 (8.5 – 14.8)	17.2 (11.7 - 25.5)	22.1 (17.0 - 30.2)	0.093

The data are presented as the median and interquartile range (IQR), or n (%). For categorical data, chi-square test was used, and for continuous variables, Kruskal-Wallis was applied. WBC: white blood cell count.

### An integrated model for the differentiation of COVID-19 from influenza and CAP

**Figure 2:**
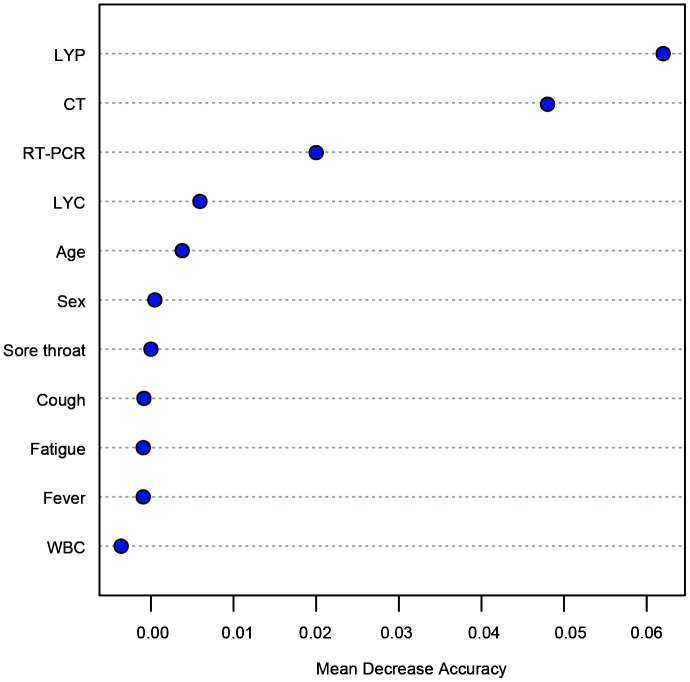
**Features ranked by mean decrease accuracy (MDA) scores in the random forest model for classification between COVID-19 and other infections.** The number of trees = 1000. LYP: lymphocyte percentage; WBC: white blood cell.

The capacity of each feature in discriminating COVID-19 from influenza and CAP was evaluated using a random forest model (decision trees = 1,000). The importance of the features was calculated based on MDA, and the top features are listed in Figure 2.

**Figure 3:**
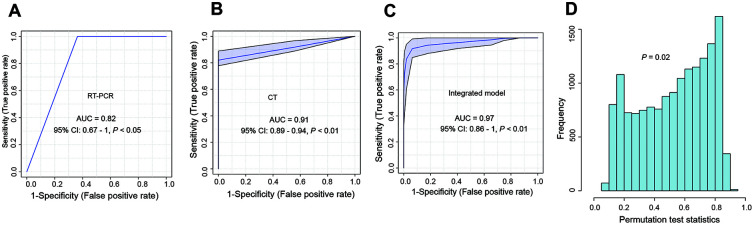
**The development of an integrated model for the differentiation of COVID-19 from other respiratory diseases in the training set.** (**A**) ROC curve for the performance of first RT-PCR; (**B**) ROC curve for the performance of CT; (**C**) ROC curve for the integrated model. The integrated model contained the results of the first RT-PCR, CT, and LYP in the blood. (**D**) The cross-validation of the integrated model using the permutation test (1000 times). ROC curve: Receiver operator characteristic curve; LYP: lymphocyte percentage.

The performance of the single and multi-feature models was assessed using ROC analysis and confusion matrices. Even though the first RT-PCR and CT alone had acceptable AUROCs (0.82 and 0.91), their F1 scores were only 0.66 and 0.67, respectively (Figure 3A, 3B and Table 3); in addition, these two models had low Matthews correlation coefficient (MCC) values (0.67 and 0.64, respectively). Next, we evaluated the performance of the combination of multiple features. Surprisingly, an integrated model with the combination of LYP, RT-PCR, and CT performed well for distinguishing COVID-19 from seasonal flu and CAP, with an AUC of 0.97 (95% CI: 0.86 - 1, *P* < 0.01) (Figure 3C). The permutation test showed that the integrated model was robust (Figure 3D). Other classification metrics, including the accuracy, sensitivity, specificity, PPV, NPV, F1 score, and MCC, were also higher than those of the models with single features.

**Table 3:** The performance of the developed models for the differentiation of COVID-19 from influenza, and community-acquired pneumonia with similar symptoms.

**Classification models**	**Model performance (Mean and 95% CI)**						
	**Accuracy (%)**	**F1 score**	**MCC**	**Sensitivity (%)**	**Specificity (%)**	**PPV (%)**	**NPV (%)**
PCR	92.0 (73.9 - 99.1)	0.66	0.67	100 (75.8 - 100)	91.3 (71.9 - 98.9)	50.1 (21.1 - 78.9)	100
CT	84.0 (63.9 - 95.5)	0.67	0.64	100 (79.8 - 100)	80.9 (58.1 - 94.6)	50.1 (29.3 - 70.7)	100
Integrated model-training set	92.0 (73.9 - 99.1)	0.81	0.78	100 (89.8 - 100)	90.5 (69.6 - 98.8)	86.7 (74.8 -92.2)	100
Integrated model-internal validation	96.0 (79.6 - 99.9)	0.86	0.85	92.1 (89.4 - 99.4)	88.2 (83.9 - 100)	92.3 (85.4 – 100)	94.5 (79.4 - 99.1)
Integrated model-external validation	92.7 (82.4 – 97.9)	0.80	0.76	88.9 (51.8 – 99.7)	93.5 (82.1 – 98.6)	72.7 (46.6 – 89.1)	97.7 (87.1 – 99.6)

The models were trained by the random forest algorithm. 95% CI: 95% confidence interval; MCC: Matthews correlation coefficient; PPV (%): positive predictive value; NPV (%): negative predictive value.

In the internal validation cohort, the multivariate model yielded an F1 score of 0.86, an MCC of 0.85, an accuracy of 96% (95% CI: 76.6 – 99.9), a sensitivity of 92% (95% CI: 89.4 -99.4) and a specificity of 88.2 (95% CI: 83.9 – 100) (Table 3).

**Figure 4:**
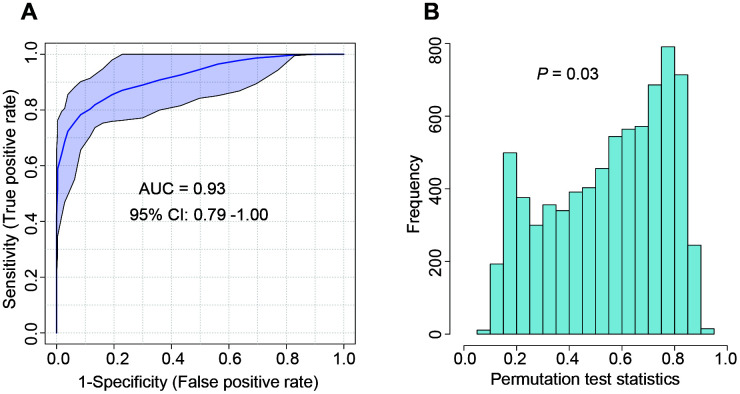
**The performance of the developed integrated model evaluated by ROC curve in the external validation cohort.** (**A**) ROC curve; (**B**) The cross-validation with the permutation test (1000 times). ROC curve: Receiver operator characteristic curve.

To further validate the feasibility of our model for distinguishing COVID-19 from seasonal flu and community-acquired pneumonia, we recruited another 55 patients from a different medical center. Similarly, the developed model performed well in this independent cohort with an F1 score of 0.80 and an MCC of 0.76 (Table 3). ROC curve analysis showed an AUROC of 0.93 (95% CI: 0.79 – 1.00) (Figure 4A). The reliability of the ROC curve prediction was validated using the permutation test (1000 times) (Figure 4B). The diagnostic accuracy was 92.7% (95% CI: 82.4 – 97.9) in the external validation cohort. The sensitivity and specificity were 88.9% (95% CI: 51.8 – 99.7), and 93.5% (95% CI: 82.1 – 98.6), respectively (Table 3).

## DISCUSSION

In this study, we successfully developed and validated a multivariate model that has the best performance for distinguishing COVID-19 from influenza and other respiratory diseases compared to current approaches. Our model is particularly useful for screening COVID-19 in regions with a low incidence rate of COVID-19 during the flu season.

We used two additional classification metrics for the characterization of the developed models, including the F1 score and MCC. The F1 score is the weighted average of precision and recall. When the dataset is unbalanced (the number of patients in one group is much larger than the number of patients in the other groups), the traditional classifier accuracy is no longer a reliable metric [8]. In contrast, the F1 score takes the data distribution into account and is a better metric for our model in this study. Additionally, the Matthews correlation coefficient (MCC) is also a reliable metric in machine learning that produces a high score only if the prediction obtained good results in all four confusion matrix categories (true positives, false negatives, true negatives, and false positives) [8].

Nucleic acid testing is influenced by the specimen collector, sample source, and timing of the acquisition, resulting in a high false negative rate. Previous studies have reported that the positive rate of throat swab RT-PCR test is only approximately 59%, and the sensitivity is 30-60% [9]. In this study, three patients tested negative by RT-PCR four times before positive results were obtained the fifth time. In contrast, our integrated model using the combination of multiple features (first PCR results, LYP, and CT images) accurately identified these COVID-19 patients.

The study was limited to a small number of COVID-19 patients due to the low incidence rate in the non-epidemic area. The majority of the patients in our study had seasonal flu and CAP. Additional validation should be considered in a more diverse demographic group than our initial cohort prior to further clinical application.

In summary, we developed an integrated multivariate model that distinguishes COVID-19 from influenza and other respiratory diseases using the random forest algorithm. Compared to the current approaches, the new model may significantly reduce the possibility of false-negative and false-positive results for COVID-19.

## MATERIALS AND METHODS

### Subjects

This multicenter study was conducted in two hospitals. The discovery and internal validation were conducted on suspected COVID-19 patients who presented to Heping Hospital Affiliated to Changzhi Medical College, Shanxi province, China, from January 23, 2020, to February 20, 2020. The external validation was conducted in Fenyang Hospital, Shanxi province, China, from March 1, 2020 to April 30, 2020. All patients underwent CT scans on the day of admission. Pharyngeal swab samples were collected for RT-PCR analysis. Blood samples were collected from each participant. The age, sex, and clinical symptoms of the patients, as well as the epidemiological characteristics of COVID-19, were also collected. A total of 50 patients were enrolled for the discovery and internal validation cohorts, and another 55 patients were included in the external validation cohort. The study protocol was approved by the Institutional Review Boards of Heping Hospital Affiliated to Changzhi Medical College (CMC-2020-1103), and Fenyang Hospital (FY-20-08). Written informed consent was obtained from each participant.

### Blood tests

Routine blood tests, including white blood cell count (WBC), lymphocyte count (LYC), and blood lymphocyte percentage (LYP) (%), were performed on the blood samples.

### Chest CT images

The CT examination was performed with a multislice spiral CT machine (TOSHIBA Aquilion 16, Japan) by two senior chest diagnostic radiologists who used a PACS workstation to read the axial images of standard 5 mm slice thickness, and 1 mm slice thickness images were used for multislice reconstruction to observe the lesions. The typical imaging manifestations of the patients were ground-glass opacification, consolidation, reticular shadow, and air bronchial sign, and most of the lesions were distributed in the subpleural areas. CT scans were read independently by two radiologists (blinded for review). Disagreements were resolved by a third experienced thoracic radiologist.

### Model construction and evaluation

We followed the guidelines for the transparent reporting of a multivariable prediction model for individual prognosis or diagnosis (TRIPOD). The analyses were performed using the randomForest package (version 4.6-14) in R 3.5.19. A total of 16 variables were analyzed for model construction including age, sex, epidemiological record, fever, sore throat, cough, fatigue, LYC, LYP, WBC, first PCR results, and chest CT imaging characteristics. The importance of the features for discriminating COVID-19 from influenza and CAP was ranked based on the mean decrease accuracy (MDA) in the random forest model (1000 trees) [10]. The more the accuracy decreases, the more important the variable. For the cohort collected from Heping Hospital Affiliated to Changzhi Medical College, after all of the 1000 trees have been grown, the biological samples that did not participate in the training of trees are used as an internal validation set to check the error rate for each tree (training/internal validation: 7:3). For external validation, all the subjects were used.

Once the ranked features were identified, several combinations of features were selected based on their ranking scores. We evaluated the performance of the models using the area under the receiver operating characteristic curve (AUROC). Classifier robustness was estimated by repeated double cross-validation (rdCV) and permutation testing 1,000 times. The accuracy, sensitivity, positive predictive value (PPV, %), negative predictive value (NPV, %), F1 score, and Matthews correlation coefficient (MCC) were also calculated. The optimal model was then validated using another set of patients.

### Ethics approval and consent to participate

The study protocol was approved by the Institutional Review Boards of Heping Hospital Affiliated to Changzhi Medical College (CMC-2020-1103), and Fenyang Hospital (FY-20-08). Written informed consent was obtained from each participant.

## References

[1] Zhu N, Zhang D, Wang W, Li X, Yang B, Song J, Zhao X, Huang B, Shi W, Lu R, Niu P, Zhan F, Ma X, et al, and China Novel Coronavirus Investigating and Research Team. A novel coronavirus from patients with pneumonia in China, 2019. N Engl J Med. 2020; 382:727–33. 10.1056/NEJMoa2001017 31978945 PMC7092803

[2] Dong E, Du H, Gardner L. An interactive web-based dashboard to track COVID-19 in real time. Lancet Infect Dis. 2020; 20:533–34. 10.1016/S1473-3099(20)30120-1 32087114 PMC7159018

[3] Wang D, Hu B, Hu C, Zhu F, Liu X, Zhang J, Wang B, Xiang H, Cheng Z, Xiong Y, Zhao Y, Li Y, Wang X, Peng Z. Clinical characteristics of 138 hospitalized patients with 2019 novel coronavirus-infected pneumonia in Wuhan, China. JAMA. 2020; 323:1061–69. 10.1001/jama.2020.1585 32031570 PMC7042881

[4] West CP, Montori VM, Sampathkumar P. COVID-19 testing: the threat of false-negative results. Mayo Clin Proc. 2020; 95:1127–29. 10.1016/j.mayocp.2020.04.004 32376102 PMC7151274

[5] Hope MD, Raptis CA, Henry TS. Chest computed tomography for detection of coronavirus disease 2019 (COVID-19): don’t rush the science. Ann Intern Med. 2020; 173:147–48. 10.7326/M20-1382 32267912 PMC7147341

[6] Tan L, Wang Q, Zhang D, Ding J, Huang Q, Tang YQ, Wang Q, Miao H. Lymphopenia predicts disease severity of COVID-19: a descriptive and predictive study. Signal Transduct Target Ther. 2020; 5:33. 10.1038/s41392-020-0148-4 32296069 PMC7100419

[7] Lebedev AV, Westman E, Van Westen GJ, Kramberger MG, Lundervold A, Aarsland D, Soininen H, Kłoszewska I, Mecocci P, Tsolaki M, Vellas B, Lovestone S, Simmons A, and Alzheimer’s Disease Neuroimaging Initiative and the AddNeuroMed consortium. Random forest ensembles for detection and prediction of Alzheimer’s disease with a good between-cohort robustness. Neuroimage Clin. 2014; 6:115–25. 10.1016/j.nicl.2014.08.023 25379423 PMC4215532

[8] Chicco D, Jurman G. The advantages of the matthews correlation coefficient (MCC) over F1 score and accuracy in binary classification evaluation. BMC Genomics. 2020; 21:6. 10.1186/s12864-019-6413-7 31898477 PMC6941312

[9] Yang Y, Yang M, Shen C, Wang F, Yuan J, Li J, Zhang M, Wang Z, Xing L, Wei J, Peng L, Wong G, Zheng H, et al Evaluating the accuracy of different respiratory specimens in the laboratory diagnosis and monitoring the viral shedding of 2019-nCoV infections. medRxiv. 2020 10.1101/2020.02.11.20021493

[10] Alam MZ, Rahman MS. A Random Forest based predictor for medical data classification using feature ranking. Inform Med Unlocked. 2019; 15:100180 10.1016/j.imu.2019.100180

